# A Search for Beta Thalassemia Trait in India

**DOI:** 10.5505/tjh.2012.21703

**Published:** 2012-12-05

**Authors:** Veda Parthasarathy

**Affiliations:** 1 Department of Pathology, ESI PGIMSR, Bangalore, India

**To the Editor,**

The beta-thalassemia trait (BTT)—or beta-thalassemia minor—is a heterozygous condition in which only a single beta-globin gene is affected. The estimated prevalence of BTT in different regions of India is reported to vary between 2.7% and 14.9% (mean: 4.5%) [1,2]. Most individuals with BTT are asymptomatic and are identified incidentally when their complete blood count (CBC) shows microcytosis [3]. Red blood cells are considered to be microcytic when the mean corpuscular volume (MCV) is <80 fL [4]. Common causes of microcytosis are iron deficiency anemia (IDA), BTT, anemia of chronic disease (ACD), lead poisoning, and sideroblastic anemia [5]; BTT must be differentiated from IDA and other causes of microcytosis. Automated red cell parameters, such as MCV, RBC count, and red cell distribution width (RDW), have been used to identify patients with a high probability of BTT [5]. The aim of the present study was to determine which routine CBC parameters would best differentiate BTT from other microcytic anemias. The study included 200 adult patients with microcytosis (MCV <80 fL). Detailed clinical history, CBC, blood smear, quantitative assessment of hemoglobin A2 (HbA2), and serum ferritin were evaluated in all cases. An HbA2 concentration >3.5% was considered diagnostic of BTT [1,6]. Iron deficiency was diagnosed based on a ferritin level <15 ng/mL [7]. Serum ferritin is the best parameter to use for IDA screening and in the absence of inflammation, a normal ferritin level generally excludes iron deficiency [3]. 

Of the 200 cases evaluated, the 39 that had an HbA2 level >3.5% constituted the BTT group (figure 1); the remaining 161 cases with an HbA2 level ≤3.5% constituted the non-BTT group. In the BTT group 3 cases had concomitant iron deficiency. In the non-BTT group 120 cases had a serum ferritin level <15 ng/mL and were diagnosed as IDA; of the remaining 41cases, 12 had associated chronic illness indicative of ACD (anemia of chronic disease) and in the other 29 the cause of microcytosis could not be identified. 

IDA was the most frequent cause of microcytosis in the present study, followed by BTT. The various red cell parameters examined in the 36 BTT cases, 120 IDA cases, and 12 ACD cases are shown in Table 1; the 3 BTT cases with concomitant iron deficiency are excluded. Mean Hb was highest in the BTT cases (10.9 g/dL) and lowest in the IDA cases (8.7 g/dL). The degree of microcytosis was most severe (62.9 fL) in the BTT cases, followed by the IDA cases (63.4 fL) and ACD cases (70 fL). The mean RBC count was highest in the BTT cases (5.7 x 10^12^/L), and slightly lower in those with IDA and ACD (4.8 x 10^12^/L). Anisocytosis (mean RDW-CV) was most common in the IDA cases (21.7%), followed by ACD (19.1%) and BTT cases (17.6%). The BTT cases had the lowest mean MCV (62.9 fL) and highest mean RBC count (5.7 x 10^12^/L). 

While evaluating microcytosis it is essential to differentiate IDA and BTT. In the present study patients with IDA had the most severe anemia and most severe degree of anisocytosis, as they had the lowest mean Hb (8.7 g/ dL) and highest mean RDW (21.7%). In contrast, the BTT group had the highest mean Hb (10.9 g/dL) and lowest mean RDW (17.6%), indicating less severe anemia and less severe anisocytosis. Shalev et al. [8] reported that the combination of a high RBC count and low MCV is characteristic of BTT. It has been suggested that the RBC count is the most efficient single test for differentiating BTT and IDA [5,9,10]. Eldibany et al. [11] reported that the RBC count, MCV, and RDW are the most useful indices for differentiating BTT and IDA. 

Controversy continues regarding the ideal red cell indices and their cut-off values for differentiating BTT and IDA. Kotwal et al. [5] conducted a study with 640 adult patients with microcytosis (MCV <80 fL), plotting receiver operator characteristic (ROC) curves and recalculating the cut-off values for the Indian setting. The cutoff values of MCV <76 fL, RBC count ≥4.9 x 10^12^/L, and RDW ≤18% were suggested to be associated with a high probability of BTT. In the present study the same cutoff values were applied to both the BTT and non-BTT groups in order to determine the sensitivity, specificity, positive predictive value (PPV), negative predictive value (NPV), and Youden’s index (Table 2). We attempted to determine the efficacy of these cutoff values for differentiating BTT from all other causes of microcytosis. 

In the present study an MCV <76 fL was observed in 94.9% (n = 34) of those in the BTT group and 34.8% (n = 56) of those in the non-BTT group. An RBC count >4.9 x 10^12^/L was observed in 76.9% (n = 27) of the BTT cases and 18.6% (n = 30) of the non-BTT cases. An RDW ≤18% was noted in 58.9% (n = 21) of the BTT cases and 9.9% (n = 16) of the non-BTT cases (Table 2). These findings indicate that MCV was the most sensitive parameter for identifying BTT; however, MCV lacks specificity when used as a single parameter [5]. Moreover, atypical carriers of BTT with a normal MCV will not be identified [9]. The RBC count and RDW have greater specificity than MCV. Youden’s index was highest for RDW, followed by the RBC count and MCV; therefore, we think a combination of MCV, RDW, and the RBC count is more effective for identifying BTT and differentiating it from other non-thalassemic microcytosis; however, it should be noted that patients with BTT and concomitant iron, vitamin B12, or folic acid deficiency, and double heterozygous δβ-thalassemics can have an elevated RDW [6,9,12]. Concomitant nutritional deficiency can also alter HbA2 levels in BTT. Microcytosis accompanied by a high RBC count and normal RDW is suggestive of BTT. These automated red cell parameters are routinely examined and offer a rapid and reliable method for BTT screening. Adequate utilization of these parameters can facilitate identification of the majority of BTT cases at no additional cost to the health care system. Identifying carriers and counseling them about the genetic implications of marrying another carrier is the most effective method for preventing beta-thalassemia major.

## Figures and Tables

**Table 1:**
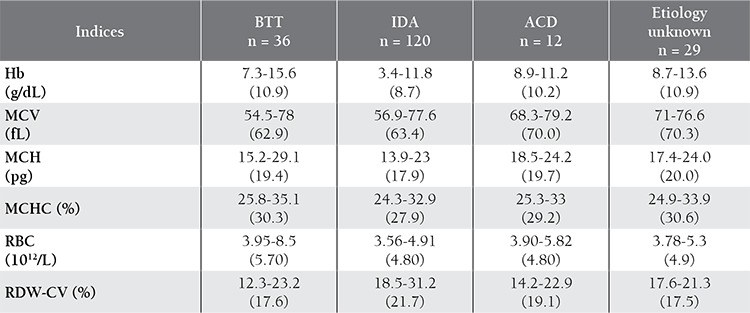
Comparison of various red cell parameters in BTT, IDA and ACD. Values within parenthesis indicate the mean.

**Table 2:**
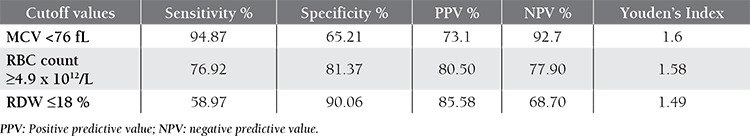
The sensitivity, specificity, PPV, NPV and Youden’s index obtained by using various parameters. Youden’s Index = sensitivity+specificity.

**Figure 1:**
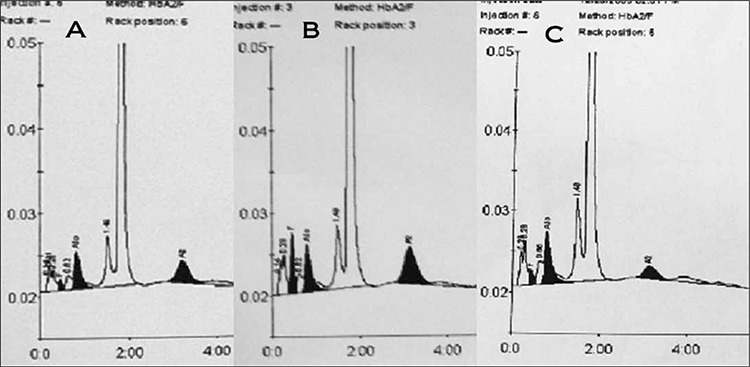
HPLC Chromatograms displaying the hemoglobin fractions obtained in BTT and non-BTT cases. A and B belong to betathalassemia carriers having HbA2 concentration of 4.5% and 6.8% respectively. C represents a normal control with HbA2 fraction of 2.3%.
